# Supplementary material for: Modeling glioblastoma heterogeneity as a dynamic network of cell states
Source: Mol Syst Biol. 2021 Sep 16;17(9):e10105. doi: 10.15252/msb.202010105 (PMC8444284; doi:10.15252/msb.202010105)
Supplement: Supplementary file 6 — Source Data for Figure 5 [file MSB-17-e10105-s004.zip › Figure5A_sourcedata/GSEA_3071/nosigdb_stateI.GseaPreranked.1622795145664/RICHARDS_ZHONG_NATURE_2018_NPCS_UPREG.html]

Details for gene set RICHARDS\_ZHONG\_NATURE\_2018\_NPCS\_UPREG[GSEA]

|  || Dataset | state13071 |
| Phenotype | NoPhenotypeAvailable |
| Upregulated in class | na\_neg |
| GeneSet | RICHARDS\_ZHONG\_NATURE\_2018\_NPCS\_UPREG |
| Enrichment Score (ES) | -0.57938486 |
| Normalized Enrichment Score (NES) | -3.121981 |
| Nominal p-value | 0.0 |
| FDR q-value | 0.0 |
| FWER p-Value | 0.0 |
Table: GSEA Results Summary

  

Fig 1: Enrichment plot: RICHARDS\_ZHONG\_NATURE\_2018\_NPCS\_UPREG      
 Profile of the Running ES Score & Positions of GeneSet Members on the Rank Ordered List

  

| PROBE | GENE SYMBOL | GENE\_TITLE | RANK IN GENE LIST | RANK METRIC SCORE | RUNNING ES | CORE ENRICHMENT || 1 | MFAP2 |  |  | 44 | 1.395 | 0.0057 | No |
| 2 | EFNB1 |  |  | 244 | 0.740 | -0.0430 | No |
| 3 | PHGDH |  |  | 279 | 0.699 | -0.0436 | No |
| 4 | TMEM98 |  |  | 510 | 0.502 | -0.1046 | No |
| 5 | IGF2BP1 |  |  | 591 | 0.457 | -0.1221 | No |
| 6 | VIM |  |  | 1105 | 0.305 | -0.2689 | No |
| 7 | RCN1 |  |  | 1250 | 0.277 | -0.3076 | No |
| 8 | BUB3 |  |  | 1687 | -0.278 | -0.4321 | No |
| 9 | KIF2C |  |  | 1735 | -0.284 | -0.4422 | No |
| 10 | CCNB2 |  |  | 1757 | -0.288 | -0.4445 | No |
| 11 | MIS18BP1 |  |  | 1764 | -0.289 | -0.4424 | No |
| 12 | APOLD1 |  |  | 1781 | -0.291 | -0.4432 | No |
| 13 | CDON |  |  | 1852 | -0.300 | -0.4598 | No |
| 14 | NOTCH1 |  |  | 1878 | -0.301 | -0.4632 | No |
| 15 | KPNA2 |  |  | 1944 | -0.312 | -0.4781 | No |
| 16 | TGIF1 |  |  | 1955 | -0.313 | -0.4769 | No |
| 17 | SNRPB |  |  | 2064 | -0.328 | -0.5043 | No |
| 18 | DEPDC1B |  |  | 2288 | -0.370 | -0.5649 | No |
| 19 | TUBB4B |  |  | 2291 | -0.370 | -0.5606 | No |
| 20 | CKS1B |  |  | 2332 | -0.380 | -0.5673 | No |
| 21 | MCM2 |  |  | 2349 | -0.383 | -0.5669 | No |
| 22 | CKAP2 |  |  | 2357 | -0.385 | -0.5638 | No |
| 23 | CENPW |  |  | 2402 | -0.393 | -0.5715 | No |
| 24 | UHRF1 |  |  | 2404 | -0.393 | -0.5666 | No |
| 25 | WDR34 |  |  | 2414 | -0.395 | -0.5639 | No |
| 26 | BUB1 |  |  | 2416 | -0.396 | -0.5589 | No |
| 27 | H2AFV |  |  | 2431 | -0.398 | -0.5577 | No |
| 28 | CKS2 |  |  | 2466 | -0.404 | -0.5623 | No |
| 29 | KIF11 |  |  | 2488 | -0.410 | -0.5630 | No |
| 30 | UCP2 |  |  | 2498 | -0.413 | -0.5602 | No |
| 31 | SPAG5 |  |  | 2519 | -0.418 | -0.5605 | No |
| 32 | CDC20 |  |  | 2540 | -0.425 | -0.5607 | No |
| 33 | PAICS |  |  | 2545 | -0.426 | -0.5562 | No |
| 34 | H2AFZ |  |  | 2572 | -0.433 | -0.5580 | No |
| 35 | DUT |  |  | 2640 | -0.450 | -0.5717 | No |
| 36 | CCNA2 |  |  | 2667 | -0.462 | -0.5732 | Yes |
| 37 | KIF22 |  |  | 2683 | -0.468 | -0.5714 | Yes |
| 38 | PAX6 |  |  | 2688 | -0.469 | -0.5663 | Yes |
| 39 | MCM6 |  |  | 2699 | -0.471 | -0.5629 | Yes |
| 40 | HAT1 |  |  | 2703 | -0.472 | -0.5575 | Yes |
| 41 | PRC1 |  |  | 2705 | -0.472 | -0.5515 | Yes |
| 42 | CENPN |  |  | 2708 | -0.474 | -0.5458 | Yes |
| 43 | MELK |  |  | 2709 | -0.474 | -0.5394 | Yes |
| 44 | KNSTRN |  |  | 2722 | -0.478 | -0.5366 | Yes |
| 45 | NDC80 |  |  | 2723 | -0.478 | -0.5302 | Yes |
| 46 | RPA3 |  |  | 2730 | -0.480 | -0.5256 | Yes |
| 47 | CENPF |  |  | 2761 | -0.490 | -0.5278 | Yes |
| 48 | NUF2 |  |  | 2781 | -0.497 | -0.5268 | Yes |
| 49 | TUBA1B |  |  | 2785 | -0.498 | -0.5210 | Yes |
| 50 | DTYMK |  |  | 2798 | -0.501 | -0.5179 | Yes |
| 51 | CDCA3 |  |  | 2829 | -0.513 | -0.5198 | Yes |
| 52 | HMGB3 |  |  | 2839 | -0.516 | -0.5156 | Yes |
| 53 | TOP2A |  |  | 2842 | -0.517 | -0.5093 | Yes |
| 54 | CKAP2L |  |  | 2857 | -0.523 | -0.5064 | Yes |
| 55 | HJURP |  |  | 2869 | -0.527 | -0.5026 | Yes |
| 56 | ANP32E |  |  | 2877 | -0.530 | -0.4976 | Yes |
| 57 | PTTG1 |  |  | 2895 | -0.540 | -0.4954 | Yes |
| 58 | SFRP1 |  |  | 2926 | -0.559 | -0.4968 | Yes |
| 59 | TACC3 |  |  | 2956 | -0.575 | -0.4976 | Yes |
| 60 | KIF23 |  |  | 2961 | -0.579 | -0.4911 | Yes |
| 61 | SMC2 |  |  | 2984 | -0.592 | -0.4896 | Yes |
| 62 | TPX2 |  |  | 3006 | -0.606 | -0.4877 | Yes |
| 63 | C19orf48 |  |  | 3013 | -0.608 | -0.4814 | Yes |
| 64 | AURKB |  |  | 3056 | -0.639 | -0.4852 | Yes |
| 65 | C21orf58 |  |  | 3078 | -0.659 | -0.4826 | Yes |
| 66 | GTSE1 |  |  | 3083 | -0.663 | -0.4749 | Yes |
| 67 | CDCA4 |  |  | 3084 | -0.663 | -0.4660 | Yes |
| 68 | TMPO |  |  | 3088 | -0.666 | -0.4580 | Yes |
| 69 | MAD2L1 |  |  | 3099 | -0.675 | -0.4519 | Yes |
| 70 | DACH1 |  |  | 3112 | -0.686 | -0.4463 | Yes |
| 71 | PRR11 |  |  | 3125 | -0.698 | -0.4405 | Yes |
| 72 | SMC4 |  |  | 3126 | -0.699 | -0.4312 | Yes |
| 73 | CENPK |  |  | 3127 | -0.700 | -0.4219 | Yes |
| 74 | MCM5 |  |  | 3132 | -0.706 | -0.4136 | Yes |
| 75 | GMNN |  |  | 3140 | -0.719 | -0.4061 | Yes |
| 76 | KIF15 |  |  | 3142 | -0.723 | -0.3967 | Yes |
| 77 | BTG3 |  |  | 3148 | -0.727 | -0.3885 | Yes |
| 78 | CDK1 |  |  | 3149 | -0.727 | -0.3788 | Yes |
| 79 | MKI67 |  |  | 3158 | -0.738 | -0.3713 | Yes |
| 80 | ORC6 |  |  | 3159 | -0.738 | -0.3614 | Yes |
| 81 | MCM3 |  |  | 3182 | -0.761 | -0.3577 | Yes |
| 82 | CDCA7 |  |  | 3203 | -0.785 | -0.3531 | Yes |
| 83 | BIRC5 |  |  | 3208 | -0.793 | -0.3437 | Yes |
| 84 | FBXO5 |  |  | 3212 | -0.797 | -0.3339 | Yes |
| 85 | RRM1 |  |  | 3215 | -0.801 | -0.3238 | Yes |
| 86 | SPC25 |  |  | 3219 | -0.806 | -0.3140 | Yes |
| 87 | MDK |  |  | 3224 | -0.811 | -0.3043 | Yes |
| 88 | UBE2C |  |  | 3225 | -0.812 | -0.2935 | Yes |
| 89 | NUSAP1 |  |  | 3237 | -0.825 | -0.2857 | Yes |
| 90 | HMGN2 |  |  | 3242 | -0.833 | -0.2757 | Yes |
| 91 | CENPM |  |  | 3269 | -0.881 | -0.2716 | Yes |
| 92 | SOX9 |  |  | 3271 | -0.883 | -0.2601 | Yes |
| 93 | CDCA5 |  |  | 3273 | -0.885 | -0.2486 | Yes |
| 94 | FEN1 |  |  | 3278 | -0.890 | -0.2379 | Yes |
| 95 | H2AFX |  |  | 3282 | -0.898 | -0.2268 | Yes |
| 96 | HELLS |  |  | 3284 | -0.901 | -0.2150 | Yes |
| 97 | ZFHX4 |  |  | 3291 | -0.915 | -0.2046 | Yes |
| 98 | CDKN3 |  |  | 3293 | -0.916 | -0.1926 | Yes |
| 99 | ZWINT |  |  | 3296 | -0.925 | -0.1809 | Yes |
| 100 | PCNA |  |  | 3319 | -0.963 | -0.1745 | Yes |
| 101 | UBE2T |  |  | 3330 | -0.979 | -0.1644 | Yes |
| 102 | MCM4 |  |  | 3341 | -1.006 | -0.1539 | Yes |
| 103 | PBK |  |  | 3354 | -1.046 | -0.1434 | Yes |
| 104 | MCM7 |  |  | 3384 | -1.147 | -0.1366 | Yes |
| 105 | GINS2 |  |  | 3386 | -1.156 | -0.1215 | Yes |
| 106 | DHFR |  |  | 3387 | -1.159 | -0.1060 | Yes |
| 107 | HMGB2 |  |  | 3390 | -1.184 | -0.0908 | Yes |
| 108 | CLSPN |  |  | 3391 | -1.184 | -0.0750 | Yes |
| 109 | CENPU |  |  | 3398 | -1.230 | -0.0603 | Yes |
| 110 | ATAD2 |  |  | 3415 | -1.278 | -0.0480 | Yes |
| 111 | RRM2 |  |  | 3420 | -1.297 | -0.0318 | Yes |
| 112 | ID4 |  |  | 3488 | -2.097 | -0.0235 | Yes |
| 113 | HIST1H4C |  |  | 3493 | -2.248 | 0.0053 | Yes |
Table: GSEA details [plain text format]

  

Fig 2: RICHARDS\_ZHONG\_NATURE\_2018\_NPCS\_UPREG: Random ES distribution      
 Gene set null distribution of ES for **RICHARDS\_ZHONG\_NATURE\_2018\_NPCS\_UPREG**
